# A robust six-gene prognostic signature for prediction of both disease-free and overall survival in non-small cell lung cancer

**DOI:** 10.1186/s12967-019-1899-y

**Published:** 2019-05-14

**Authors:** Shuguang Zuo, Min Wei, Hailin Zhang, Anxian Chen, Junhua Wu, Jiwu Wei, Jie Dong

**Affiliations:** 1 Jiangsu Key Laboratory of Molecular Medicine, Medical School of Nanjing University, Nanjing, 210093 China; 2 Center for Translational Medicine, Huaihe Hospital of Henan University, Kaifeng, 475001 Henan Province China; 3 Nanjing University Hightech Institute at Suzhou, Suzhou, 215123 China

**Keywords:** Non-small cell lung cancer, Disease-free survival, Overall survival, Risk score, Prognostic signature

## Abstract

**Background:**

The high mortality of patients with non-small cell lung cancer (NSCLC) emphasizes the necessity of identifying a robust and reliable prognostic signature for NSCLC patients. This study aimed to identify and validate a prognostic signature for the prediction of both disease-free survival (DFS) and overall survival (OS) of NSCLC patients by integrating multiple datasets.

**Methods:**

We firstly downloaded three independent datasets under the accessing number of GSE31210, GSE37745 and GSE50081, and then performed an univariate regression analysis to identify the candidate prognostic genes from each dataset, and identified the gene signature by overlapping the candidates. Then, we built a prognostic model to predict DFS and OS using a risk score method. Kaplan–Meier curve with log-rank test was used to determine the prognostic significance. Univariate and multivariate Cox proportional hazard regression models were implemented to evaluate the influences of various variables on DFS and OS. The robustness of the prognostic gene signature was evaluated by re-sampling tests based on the combined GEO dataset (GSE31210, GSE37745 and GSE50081). Furthermore, a The Cancer Genome Atlas (TCGA)-NSCLC cohort was utilized to validate the prediction power of the gene signature. Finally, the correlation of the risk score of the gene signature and the Gene set variation analysis (GSVA) score of cancer hallmark gene sets was investigated.

**Results:**

We identified and validated a six-gene prognostic signature in this study. This prognostic signature stratified NSCLC patients into the low-risk and high-risk groups. Multivariate regression and stratification analyses demonstrated that the six-gene signature was an independent predictive factor for both DFS and OS when adjusting for other clinical factors. Re-sampling analysis implicated that this six-gene signature for predicting prognosis of NSCLC patients is robust. Moreover, the risk score of the gene signature is correlated with the GSVA score of 7 cancer hallmark gene sets.

**Conclusion:**

This study provided a robust and reliable gene signature that had significant implications in the prediction of both DFS and OS of NSCLC patients, and may provide more effective treatment strategies and personalized therapies.

**Electronic supplementary material:**

The online version of this article (10.1186/s12967-019-1899-y) contains supplementary material, which is available to authorized users.

## Background

Lung cancer is the leading cause of cancer death worldwide, and non-small cell lung cancer (NSCLC) composes the majority (approximately 85%) of all lung cancers [1, 2]. Despite advances in treatment strategies, the high mortality rate for lung cancer patients has not considerably declined, due to the late diagnosis of the disease [3]. The major clinical determinants of NSCLC prognosis include tumor extension, performance status and histological type [4, 5]. However, various disease outcomes have been identified in patients with similar clinical and pathological features, suggesting that the current clinical prognostic factors used may be insufficient to consistently predict individual clinical outcomes [6]. This emphasizes the necessity of identifying robust and reliable prognostic markers with higher sensitivity and accuracy in NSCLC.

Transcriptome profiling has widely been used to characterize prognostic signatures in patients with lung cancer, and has generated a number of candidate biomarkers with potential clinical values [7–9]. However, the suggested signatures lack consistency among studies and provide limited prognostic information, partially due to the limited sample size and technical factors. Moreover, NSCLC is a highly heterogeneous disease, thus it is critical to identify a reliable signature that can define patients who are at a high-risk to develop disease recurrence. To this end, integrating the results from multiple studies holds promise for more robust prognostic signatures. In addition, most investigations used overall survival (OS) rather than tumor recurrence as an end point [9–11]. Disease-free survival (DFS) is defined as the interval from surgery to the first diagnosis of any type of relapse or death, and is used as a possible alternative for OS.

Therefore, we attempted to identify and validate a robust and reliable prognostic signature for DFS and OS prediction by integrating multiple datasets of NSCLC patients. In the present study, we revealed a six-gene signature with a reliable prognostic value in NSCLC, which might complement conventional clinical prognostic factors, and further provide more effective therapeutic interventions and personalized therapies for NSCLC patients.

## Methods

### Patient data

Gene expression data and corresponding clinical information data of NSCLC patients were obtained from the publicly available database Gene Expression Omnibus (GEO, http://www.ncbi.nlm.nih.gov/geo/). Three independent datasets were collected in this study, under the accessing number of GSE31210 [12, 13], GSE37745 [14] and GSE50081 [15]. These gene expression data were generated using the same chip platform Affymetrix HG-U133 Plus 2.0 platform. GSE31210 consisted of a total of 226 lung adenocarcinoma cases. GSE37745 included 196 NSCLC cases, including 106 adenocarcinoma, 24 large cell carcinoma, and 66 squamouscarcinoma. GSE50081 included 181 NSCLC cases, including 127 adenocarcinoma, 7 large cell carcinoma, 43 squamous carcinoma, and 4 adenosquamous carcinoma. A total of 603 cases were enrolled for OS analysis, including 226 patients from GSE31210, 196 patients from GSE37745, and 181 patients from GSE50081. For DFS analysis, a total of 499 patients were finally included, including 226 patients from GSE31210, 96 patients from GSE37745, and 177 patients from GSE50081. The details of the patients’ clinical information in each dataset are described in Tables 1 and 2. All microarray data were normalized using robust multi-array average (RMA) and microarray Suite 5 (MAS5) methods, and log2-scale transformed in this study.

**Table 1:** Univariate and multivariate Cox regression analysis of the gene signature and disease-free survival of NSCLC patients

Variables		Patients (N)	Univariate analysis		Multivariate analysis	
			HR (95% CI)	*P*	HR (95% CI)	*P*
GSE31210						
Age	≤ 65/> 65	176/50	1.89 (1.12–3.18)	1.70E−02	2.36 (1.37–4.04)	1.83E−03
Gender	Female/male	121/105	1.27 (0.78–2.07)	3.38E−01		
ALK fusion	+/−	215/11	0.74 (0.18–3.02)	6.73E−01		
EGFR mutation	+/−	99/127	0.60 (0.37–0.98)	4.29E−02	1.98 (0.88–4.47)	1.00E−01
KRAS mutation	+/−	206/20	0.98 (0.42–2.26)	9.56E−01		
Triple negative	No/yes	158/68	1.87 (1.14–3.08)	1.39E−02	2.91 (1.30–6.54)	9.71E−03
Myc copy	Low/high	207/17	1.04 (0.42–2.59)	9.33E−01		
Stage	I/II	168/58	3.16 (1.92–5.21)	6.09E−06	2.77 (1.63–4.71)	1.62E−04
Smoking	No/yes	115/111	1.33 (0.82–2.18)	2.52E−01		
Risk score	Low/high	123/103	3.26 (1.92–5.53)	1.24E−05	3.10 (1.77–5.41)	7.17E−05
GSE37745						
Age	≤ 65/> 65	45/51	0.89 (0.50–1.55)	6.70E−01		
Gender	Female/male	46/50	1.01 (0.58–1.78)	9.60E−01		
Stage	I/II	63/18	1.13 (0.54–2.38)	7.45E−01		
Stage	I/III	63/14	1.78 (0.81–3.91)	1.48E−01		
Stage	I/IV	63/1	5.71 (0.75–43.63)	9.32E−02		
Histological type	Adeno/large	53/13	1.05 (0.46–2.42)	9.08E−01		
Histological type	Adeno/squamous	53/30	1.12 (0.60–2.10)	7.17E−01		
WHO performance status	0/1	54/32	2.21 (1.21–4.03)	1.02E−02	2.43 (1.32–4.47)	4.31E−03
WHO performance status	0/2	54/6	1.04 (0.25–4.42)	9.52E−01	1.12 (0.26–4.75)	8.77E−01
WHO performance status	0/3	54/4	1.66 (0.50–5.53)	4.08E−01	1.62 (0.49–5.38)	4.34E−01
Risk score	Low/high	46/50	2.31 (1.27–4.21)	6.08E−03	2.49 (1.36–4.55)	3.10E−03
GSE50081						
Age	≤ 65/> 65	59/118	1.24 (0.69–2.24)	4.77E−01		
Gender	Female/male	81/96	1.68 (0.95–2.99)	7.59E−02		
Stage	I/II	124/53	1.87 (1.06–3.28)	3.03E−02	1.71 (0.97–3.01)	6.30E−02
Histological type	Adeno/large	124/7	1.04 (0.25–4.31)	9.59E−01		
Histological type	Adeno/squamous	124/42	0.73 (0.10–5.36)	7.61E−01		
Histological type	Adeno/other	124/4	0.74 (0.38–1.45)	3.79E−01		
Smoking	No/yes	24/133	0.70 (0.35–1.40)	3.14E−01		
Risk score	Low/high	83/94	2.42 (1.33–4.43)	4.02E−03	2.30 (1.26–4.22)	6.97E−03
All patients						
Age	≤ 65/> 65	280/219	1.36 (1.00–1.84)	5.04E−02		
Gender	Female/male	248/251	1.31 (0.96–1.78)	8.51E−02		
Stage	I/II	355/129	2.05 (1.47–2.85)	2.02E−05	1.72 (1.23–2.40)	1.50E−03
Stage	I/III	355/14	3.45 (1.68–7.11)	7.74E−04	2.62 (1.27–5.43)	9.41E−03
Stage	I/IV	355/1	22.26 (3.01–164.44)	2.36E−03	14.79 (2.00–109.7)	8.40E−03
Histological type	Adeno/large	403/20	1.48 (0.75–2.93)	2.61E−01		
Histological type	Adeno/squamous	403/72	0.76 (0.11–5.44)	7.85E−01		
Histological type	Adeno/other	403/4	1.17 (0.77–1.78)	4.52E−01		
Risk score	Low/high	252/247	2.7 (1.94–3.76)	4.16E−09	2.39 (1.70–3.35)	4.47E−07

HR: hazard ratio; CI: confidence interval; Adeno: adenocarcinoma; Large: large cell carcinoma; Squamous: squamous cell carcinoma

**Table 2:** Univariate and multivariate Cox regression analysis of the gene signature and overall survival of NSCLC patients

Variables		Patients (N)	Univariate analysis		Multivariate analysis	
			HR (95% CI)	*P*	HR (95% CI)	*P*
GSE31210						
Age	≤ 65/> 65	176/50	2.58 (1.31–5.08)	5.99E−03	3.51 (1.72–7.15)	5.44E−04
Gender	Female/male	121/105	1.52 (0.78–2.96)	2.19E−01		
ALK fusion	+/−	215/11	1.49 (0.36–6.24)	5.82E−01		
EGFR mutation	+/−	99/127	0.47 (0.24–0.93)	2.96E−02	1.87 (0.65–5.37)	2.47E−01
KRAS mutation	+/−	206/20	0.87 (0.27–2.85)	8.17E−01		
Triple negative	No/yes	158/68	2.19 (1.12–4.26)	2.12E−02	2.95 (1.06–8.19)	3.76E−02
Myc copy	Low/high	207/17	0.7 (0.17–2.9)	6.18E−01		
Stage	I/II	168/58	4.23 (2.17–8.24)	2.17E−05	3.45 (1.72–6.93)	5.06E−04
Smoking	No/yes	115/111	1.64 (0.84–3.2)	1.50E−01		
Risk score	Low/high	129/97	6.14 (2.68–14.07)	1.84E−05	5.47 (2.3–12.99)	1.21E−04
GSE37745						
Age	≤ 65/> 65	102/94	1.35 (0.98–1.88)	6.88E−02		
Gender	Female/male	89/107	1.1 (0.79–1.52)	5.85E−01		
Stage	I/II	130/35	1.22 (0.79–1.87)	3.66E−01	1.14 (0.73–1.77)	5.68E−01
Stage	I/III	130/27	1.86 (1.19–2.93)	6.88E−03	1.63 (1.02–2.63)	4.27E−02
Stage	I/IV	130/4	1.31 (0.42–4.15)	6.43E−01	1.97 (0.61–6.35)	2.57E−01
Histological type	Adeno/large	106/24	0.89 (0.52–1.53)	6.75E−01		
Histological type	Adeno/squamous	106/66	1.26 (0.88–1.79)	2.05E−01		
WHO performance status	0/1	105/75	1.91 (1.35–2.7)	2.38E−04	1.96 (1.39–2.79)	1.53E−04
WHO performance status	0/2	105/12	1.95 (1.01–3.79)	4.83E−02	1.81 (0.92–3.57)	8.66E−02
WHO performance status	0/3	105/4	1.13 (0.36–3.61)	8.32E−01	1.09 (0.34–3.54)	8.80E−01
Risk score	Low/high	97/99	1.56 (1.12–2.16)	8.31E−03	1.44 (1.02–2.04)	3.80E−02
GSE50081						
Age	≤ 65/> 65	59/122	1.56 (0.93–2.61)	9.04E−02		
Gender	Female/male	83/98	1.93 (1.19–3.14)	7.80E−03	2 (1.23–3.26)	5.47E−03
Stage	I/II	127/54	1.69 (1.05–2.72)	3.10E−02	1.63 (1.01–2.63)	4.71E−02
Histological type	Adeno/large	127/7	1.71 (0.62–4.74)	3.01E−01		
Histological type	Adeno/squamous	127/43	1.84 (0.57–5.92)	3.03E−01		
Histological type	Adeno/other	127/4	0.8 (0.46–1.39)	4.35E−01		
Smoking	No/yes	24/136	1.39 (0.66–2.92)	3.89E−01		
Risk score	Low/high	79/102	2.21 (1.34–3.64)	1.81E−03	2.09 (1.27–3.45)	3.91E−03
All patients						
Age	≤ 65/> 65	337/266	1.92 (1.5–2.46)	2.48E−07	1.83 (1.42–2.35)	2.94E−06
Gender	Female/male	293/310	1.45 (1.13–1.86)	3.79E−03	1.27 (0.98–1.64)	7.14E−02
Stage	I/II	425/147	1.67 (1.26–2.21)	3.43E−04	1.5 (1.13–2)	5.31E−03
Stage	I/III	425/27	3.6 (2.34–5.56)	7.02E−09	2.79 (1.79–4.36)	6.58E−06
Stage	I/IV	425/4	2.53 (0.81–7.93)	1.12E−01	3.23 (1.02–10.23)	4.58E−02
Histological type	Adeno/large	459/31	1.76 (1.1–2.81)	1.83E−02	1.51 (0.94–2.43)	9.16E−02
Histological type	Adeno/squamous	459/109	2.29 (0.73–7.18)	1.56E−01	1.92 (0.6–6.1)	2.70E−01
Histological type	Adeno/other	459/4	1.85 (1.39–2.45)	2.09E−05	1.27 (0.94–1.72)	1.18E−01
Risk score	Low/high	305/298	2.11 (1.63–2.72)	1.03E−08	1.65 (1.26–2.18)	3.32E−04

HR: hazard ratio; CI: confidence interval; Adeno: adenocarcinoma; Large: large cell carcinoma; Squamous: squamous cell carcinoma

The genomic data and clinical information of NSCLC patients in The Cancer Genome Atlas (TCGA) were obtained from the University of California Santa Cruz Xenabrowser (UCSC Xena, http://xena.ucsc.edu/) [16]. This cohort has 761 NSCLC patients with the corresponding gene expression data (read count) and clinical information (including survival data).

### Prognostic gene signature screening

**Fig. 1:**
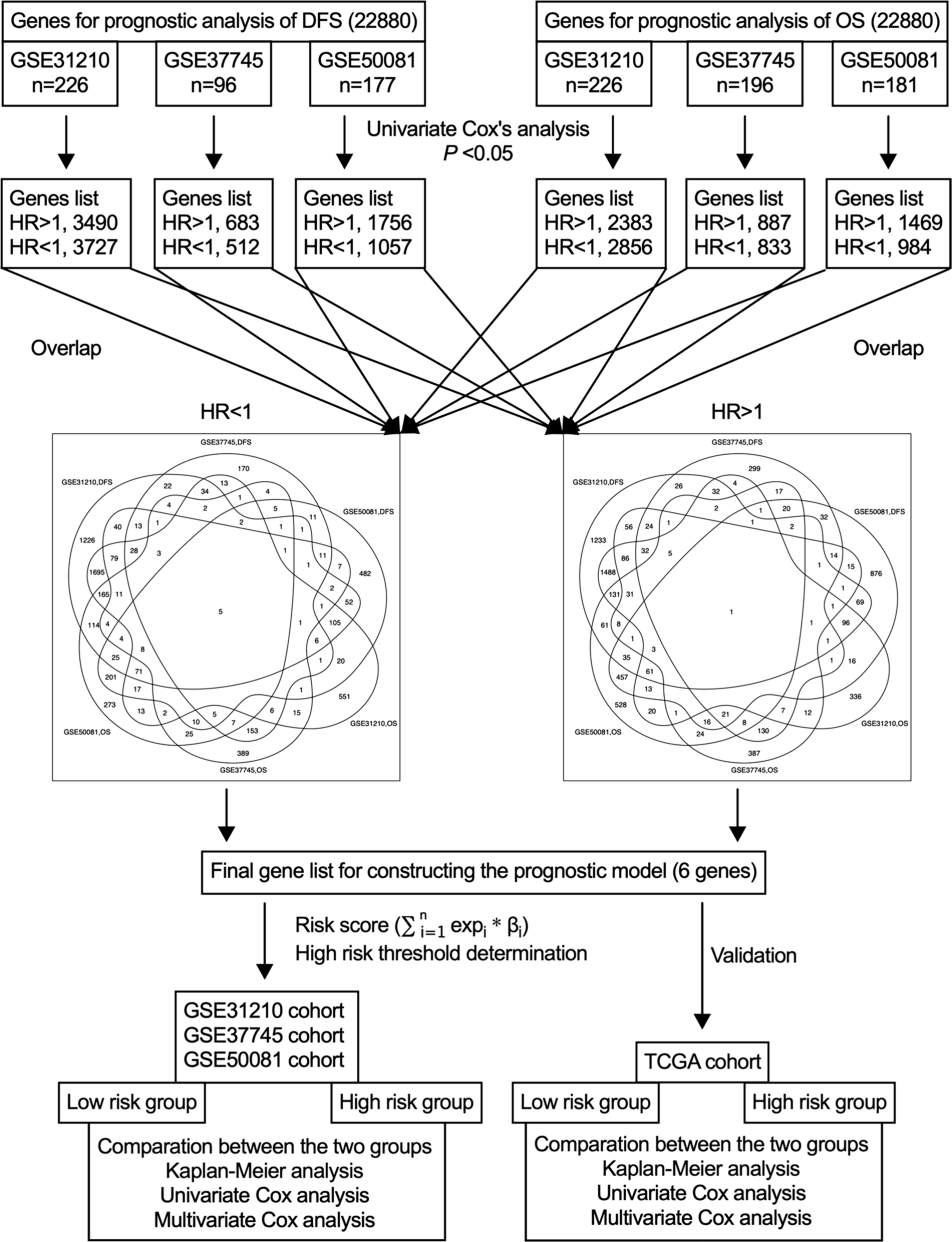
Flow diagram of this study

In this study, we firstly screened candidate prognostic genes from each cohort, and selected the common ones for constructing the prognostic gene signature. Then, the prognostic value of the signature was validated using each cohort. The flow diagram of this study is illustrated in Fig. 1. An univariate Cox proportional hazard regression model was implemented to determine the association of gene expression with DFS and OS in each cohort. Genes under a cutoff value of P < 0.05 were defined as candidate genes related to OS and DFS, and the common genes among three datasets were selected to construct the prognostic signature. Hazard ratio (HR) from the univariate Cox regression analysis was used to determine the protective (HR < 1) and risky genes (HR > 1).

Then, a risk score was established for each patient by calculating the expression values of the selected genes weighted by regression coefficients in the univariate Cox regression analysis. The formula used was as follows:

$$Risk score = \mathop \sum \limits_{i = 1}^{n} exp_{i} *\beta_{i}$$

Where n is the number of selected genes, exp_i_ is the expression level of gene i, and β_i_ represents the regression coefficient of gene i. Subsequently, the risk score was dichotomized at the median value, and patient whose risk score was greater than the median value was divided into a high-risk group, otherwise into alow-risk group.

### Evaluation of the robustness of prognostic gene signature by re-sampling tests

The data from three GEO datasets (GSE31210 [12, 13], GSE37745 [14] and GSE50081 [15]) were combined. Then a subset that contained 70% samples of the combined GEO dataset (re-sampling) was randomly selected and used to determine the prediction power of the gene signature for DFS and OS of the NSCLC patients. This re-sampling test was repeated 100 times.

### Association of prognostic gene signature and cancer hallmarks

A total of 50 hallmark gene sets which are currently recognized were downloaded from the molecular signature database (MSigDB, http://software.broadinstitute.org/gsea/msigdb). Next, gene set variation analysis (GSVA) package and its ssGSEA method (http://www.bioconductor.org) were implemented for these 50 hallmark gene sets to further obtain the GSVA scores of each gene set for each sample in the combined GEO datasets [17]. The GSVA score devotes the degree of absolute enrichment of a gene set in each sample. After that, Pearson’s correlation analysis was performed to investigate whether the GSVA score of the members of the given gene set was correlated with the risk score. The correlation coefficients (R), confidence interval (CI) and P values were calculated.

### Statistical analysis

The DFS and OS were calculated using Kaplan–Meier curves, and the statistical difference was determined by log-rank test. Influences of various variables on DFS and OS were evaluated by univariate and multivariate Cox proportional hazard regression models. HR and the 95% CI were generated using Cox proportional hazards models. The receiver operating characteristic (ROC) curve analysis was carried out to compare the predictive accuracy of the gene signature. A P value < 0.05 was set as the statistically as the significant difference.

## Results

### Identification of a six-gene prognostic signature

We firstly identified survival-related genes using univariate Cox regression analysis in each dataset. Under the cut-off threshold of Cox P < 0.05, 7217 genes in GSE31210, 1195 genes in GSE37745 and 2813 genes in GSE50081 were identified as candidate predictive genes that presented close association with DFS. Similarly, 2539 genes in GSE31210, 1720 genes in GSE37745 and 2453 genes in GSE50081 were identified to be involved in OS. By overlapping these candidate genes among three datasets, a set of 6 common genes was screened finally, including one risky gene (HR > 1) and 5 protective genes (HR < 1). The general information of these 6 genes is displayed in Table 3.

**Table 3:** Overall information of the 6 genes for constructing the prognostic signature

Gene stable ID	Gene name	Gene type	Chromosome	Gene start (bp)	Gene end (bp)
ENSG00000152527	PLEKHH2	Protein coding	2	43,637,273	43,767,987
ENSG00000136003	ISCU	Protein coding	12	108,562,582	108,569,384
ENSG00000079101	CLUL1	Protein coding	18	596,988	650,334
ENSG00000101938	CHRDL1	Protein coding	X	110,673,856	110,795,819
ENSG00000124374	PAIP2B	Protein coding	2	71,182,739	71,227,083
ENSG00000163814	CDCP1	Protein coding	3	45,082,278	45,146,422

### The six-gene signature predicts survival of NSCLC patients

**Fig. 2:**
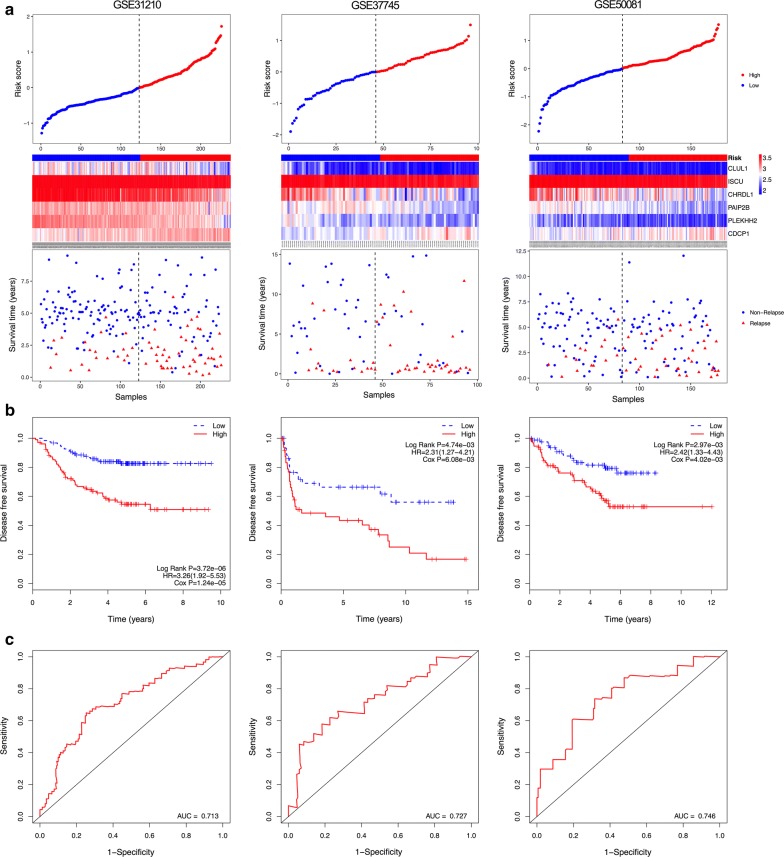
Correlation between the six-gene signature and the disease-free survival (DFS) of patients in three datasets. **a** The distribution of risk scores, gene expression levels and patient relapse status. **b** Kaplan–Meier curves of DFS of the low- and high-risk groups. **c** ROC curve for the 5-year survival prediction by the six-gene signature. The black dotted line in **a** represents the median risk score cut-off dividing patients into low- and high-risk groups

According to the gene expression and regression coefficients of the 6 genes, a prognostic model was developed to predict prognosis using a risk score method. In the prognostic model, each patient was endowed a risk score. Using the median risk score value as the cut-off point, patients in each dataset were classified into low-risk and high-risk groups. The DFS prediction power of the six-gene signature for patients in each dataset is displayed in Fig. 2. The distribution of gene risk scores, gene expression levels, and patients’ relapse status in each dataset are shown in Fig. 2a.

Kaplan–Meier curves showed that patients in the high-risk groups presented significantly shorter DFS than those in the low-risk groups (GSE31210: HR = 3.26, 95% CI 1.92–5.53, P < 0.05; GSE37745: HR = 2.31, 95% CI 1.27–4.21, P < 0.05; GSE50081: HR = 2.42, 95% CI 1.33–4.43, P < 0.05) (Fig. 2b). Furthermore, a time-dependent ROC curve was performed to evaluate the sensitivity and specificity of the six-gene signature for DFS prediction. Notably, the six-gene signature achieved AUC values of 0.713 in GSE31210, 0.727 in GSE37745 and 0.746 in GSE50081 (Fig. 2c), suggesting a substantially effective performance for DFS prediction.

**Fig. 3:**
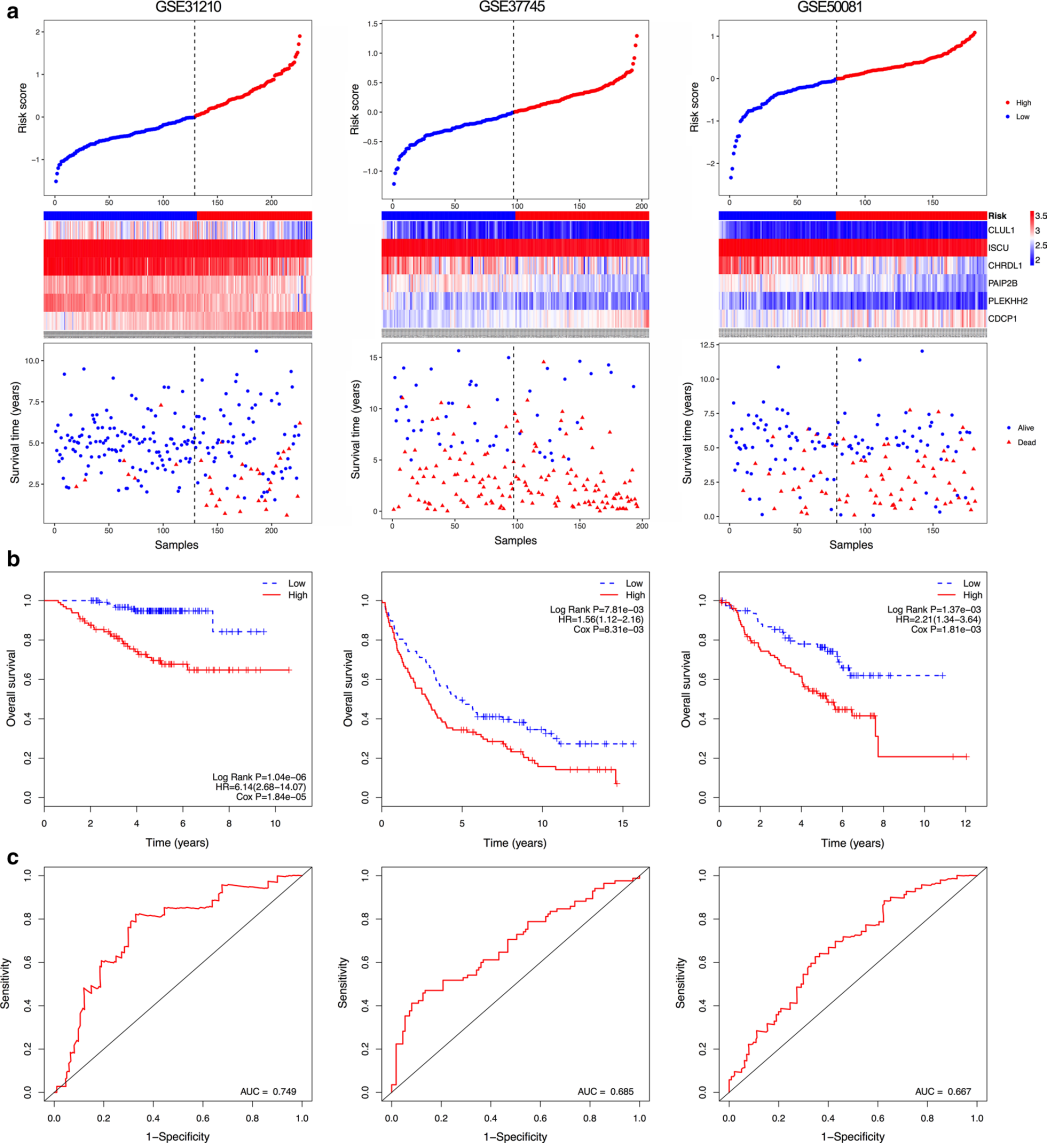
Correlation between the six-gene signature and overall survival (OS) of patients in three datasets. **a** The distribution of risk scores, gene expression levels and patient survival status. **b** Kaplan–Meier curves of OS of low- and high-risk groups. **c** ROC curve for the 5-year survival prediction by the six-gene signature. The black dotted line in **a** represents the median risk score cut-off dividing patients into low- and high-risk groups

The OS prediction value of the six-gene signature for patients in each dataset is shown in Fig. 3. Figure 3a illustrates the distribution of gene risk scores, gene expression levels and patients’ survival status in each dataset. Consistent with our previous finding, patients in the high-risk groups had significantly shorter OS when compared with those in the low-risk groups (GSE31210: HR = 6.14, 95% CI 2.68–14.07, P < 0.05; GSE37745: HR = 1.56, 95% CI 1.12–2.16, P < 0.05; GSE50081: HR = 2.21, 95% CI 1.34–3.64, P < 0.05) (Fig. 3b). Patients with high risk scores tended to have poorer clinical outcomes compared with those with low risk scores. In addition, the time-dependent ROC curve was implemented to measure the sensitivity and specificity of the six-gene signature for OS prediction in each dataset. Markedly, the signature achieved AUC values of 0.749, 0.685 and 0.667 in GSE31210, GSE37745 and GSE50081, respectively (Fig. 3c), implying a high OS prediction performance.

### The six-gene prognostic signature is robust

Previous study has demonstrated that tumor heterogeneity limits the generation of robust prognostic biomarker [18]. Thus, we conducted re-sampling tests for validation of the robustness of the prognostic gene signature. As shown in Additional file 1: Table S1, we found that in all the random 100 model validations of prediction power of the gene signature for OS by re-sampling, the P values were less than 0.0001 in each univariate Cox and Kaplan–Meier analysis. Notably, the six-gene signature achieved the AUC values of more than 0.650 for 1, 2, 3, 4, and 5-year OS in the combined GEO datasets, demonstrating a high OS prediction performance. Similarly, among all the random 100 model validations of prediction power of the gene signature for DFS, the P values were less than 0.0001 in each univariate Cox and Kaplan–Meier analysis (Additional file 2: Table S2). Moreover, the six-gene signature obtained the AUC values of more than 0.610 for 1, 2, 3, 4, and 5-year DFS in the combined GEO datasets (Additional file 2: Table S2), which implicates that this signature has an effective performance for DFS prediction. Overall, these results suggest this six-gene signature for predicting prognosis of NSCLC patients is robust.

### The six-gene signature is an independent prognostic factor

Here, we performed univariate and multivariate Cox regression models in these three datasets. The six-gene risk score and other clinicopathological factors, including age, gender, stage, histological type, gene mutation, smoking and performance status were used as covariates. The association between these factors and DFS is shown in Table 1. Univariate regression analysis indicated that age, EGFR mutation, triple negative status, disease stage and risk score were significantly associated with the DFS of NSCLC patients in GSE31210; WHO performance status and risk score were significantly associated with the DFS of patients in GSE37745; and stage and risk score were related to the DFS of patients in GSE50081. In the entire cohort, stage and risk score were identified to have significant correlation with the DFS of NSCLC patients. Moreover, in order to determine whether the six-gene signature was independent of other clinical factors, we performed a multivariate regression analysis, and found a significant correlation of the six-gene signature with DFS in three datasets (GSE31210: HR = 3.10, 95% CI 1.77–5.41, P = 7.17E−05; GSE37745: HR = 2.49, 95% CI 1.36–4.55, P = 3.10E−03; GSE50081: HR = 2.30, 95% CI 1.26–4.22, P = 6.97E−03) and the entire cohort (HR = 2.39, 95% CI = 1.70−3.35, P = 4.47E−07),after adjusting for other clinical factors. The result indicated that the six-gene risk score was an independent adverse DFS factor for NSCLC patients.

The correlation of risk score and other clinicopathological factors with the OS of NSCLC patients is shown in Table 2. We performed an univariate regression analysis to determine the correlation between these factors and OS. Our results indicated that age, EGFR mutation, triple negative status, stage and risk score were OS prognostic factors for NSCLC patients in GSE31210; stage I/III, WHO performance status and risk score were significantly related to OS of patients in GSE37745; and age, stage and risk score were OS prognostic factors for patients in GSE50081. In the entire cohort, age, gender, stage and risk score were correlated with OS of NSCLC patients. Subsequent multivariate regression analysis indicated that the six-gene signature was an independent OS prognostic factor in three datasets (GSE31210: HR = 5.47, 95% CI 2.30–12.99, P = 1.21E−04; GSE37745: HR = 1.44, 95% CI 1.02–2.04, P = 3.80E−02; GSE50081: HR = 2.09, 95% CI 1.27–3.45, P = 3.91E−03) and entire cohort (HR = 1.65, 95% CI 1.26–2.18, P = 3.32E−04), after adjusting for other clinical factors. Taken together, our data show that the six-gene risk score was an independent adverse prognostic factor for both DFS and OS of NSCLC patients.

**Fig. 4:**
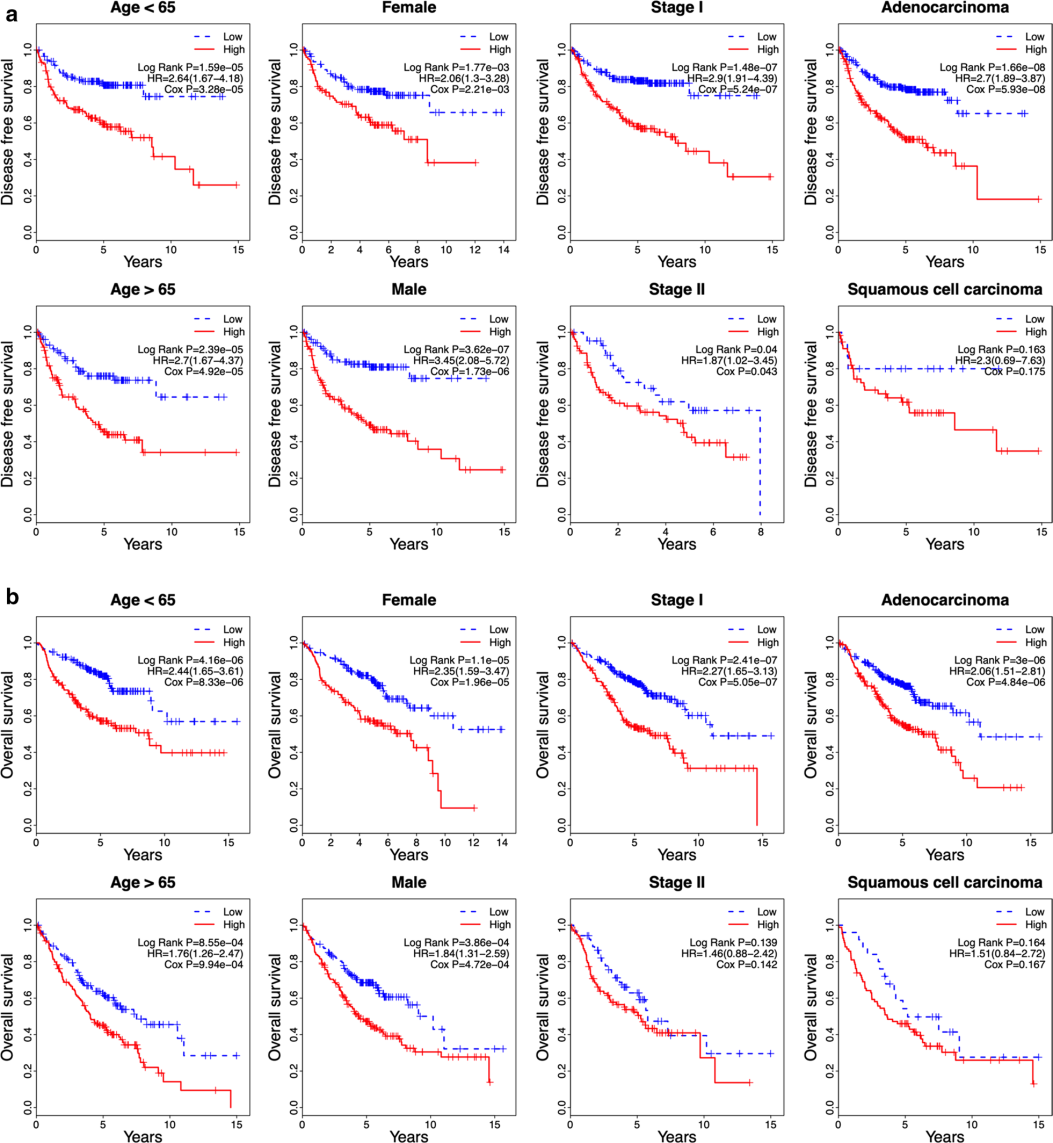
Kaplan–Meier analysis of DFS and OS for NSCLC patients stratified by age, gender, stage and histological type

Furthermore, we performed a data stratification analysis on the entire cohort. These patients (499 patients for DFS and 603 for OS) were factitiously stratified based on their clinical parameters, such as age (≤ 65/> 65), gender (female/male), stage (I/II) and histological type (adenocarcinoma/squamous carcinoma). Because of the small sample size, patients in stage III and IV, and patients with large cell cancer were removed from the stratification analysis. The results showed that the six-gene risk score remained the ability of predicting the DFS and OS within each stratum. In Fig. 4a, the results of stratification analysis showed that high-risk patients in each stratum of age, gender and early stage had significantly shorter DFS than low-risk patients (P < 0.05). For patients with adenocarcinoma, high-risk patients showed significantly shorter DFS than low-risk patients (P < 0.05), while there was no significant difference between high-risk and low-risk patients for patients with squamous carcinoma, might due to the small sample size of patients with squamous carcinoma. In Figure 4b, the results of stratification analysis indicated that high-risk patients in each stratum presented significantly poorer OS than low-risk patients (P < 0.05), except for patients in stage II and patients with squamous carcinoma. Taken together, our findings suggested that the six-gene signature was independent of other clinical features for DFS and OS prediction in NSCLC patients.

### Further validation of the six-gene signature using another independent dataset

**Fig. 5:**
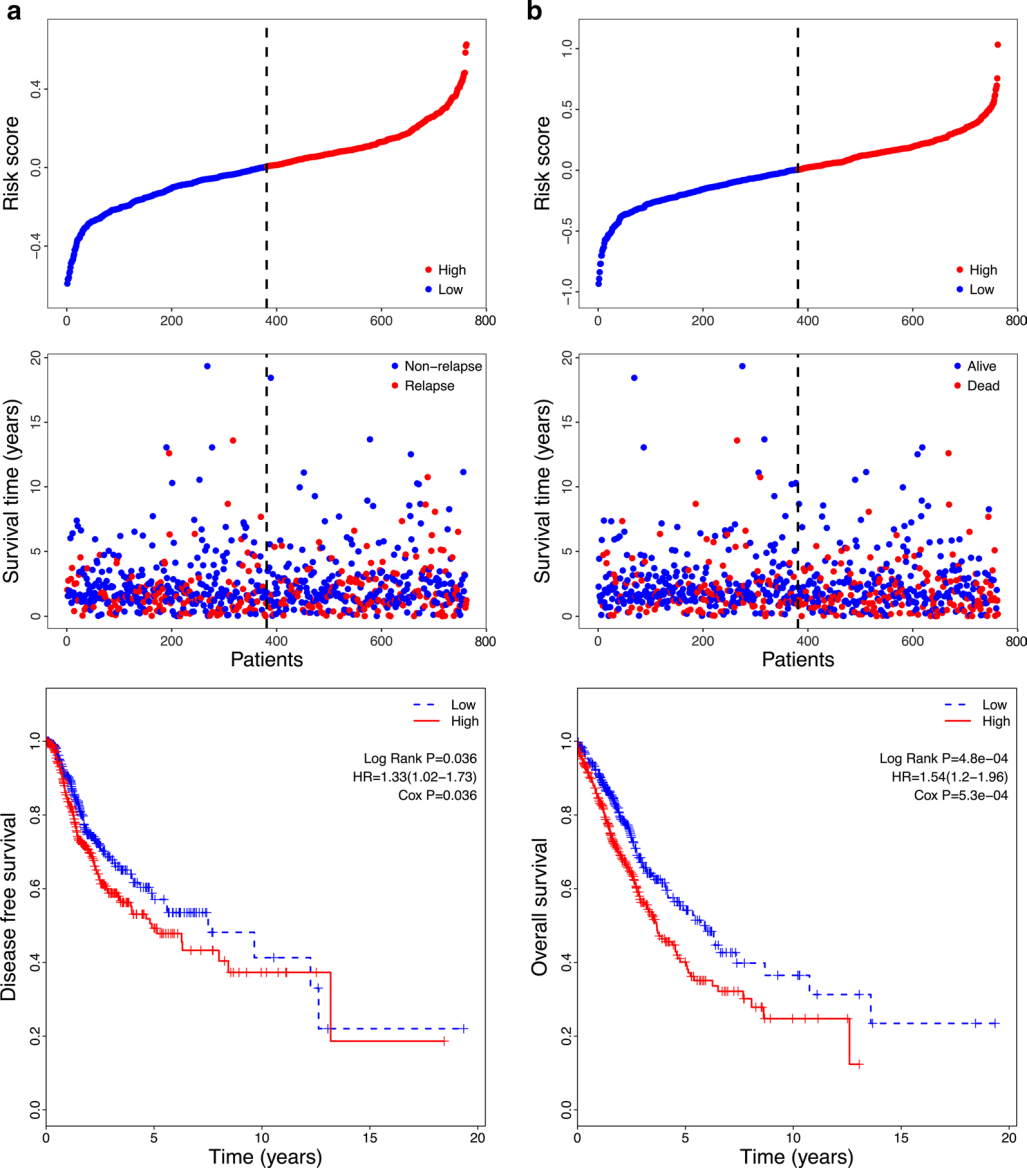
Correlation between the six-gene signature and DFS/OS of patients in the TCGA dataset. **a** The distribution of risk scores, patient relapse status, and Kaplan–Meier curves of DFS of low- and high-risk groups. **b** The distribution of risk scores, patient survival condition, and Kaplan-Meier curves of OS of low- and high-risk groups

To investigate the reliability of the six-gene signature, another independent dataset from the TCGA was used for further validation. The risk score of each sample in this dataset was calculated, and the samples were then classified into low- and high-risk groups using the median risk score value as the cut-off point (Fig. 5a, b). Kaplan–Meier and univariate Cox regression analysis exhibited that the patients in the high-risk group had obviously shorter DFS than those in the low-risk groups (HR = 1.33, 95% CI 1.02–1.73, P < 0.05) (Fig. 5a and Table 4). Similarly, patients in the high-risk group presented remarkably shorter OS compared to those in the low-risk groups (HR = 1.54, 95% CI 1.20–1.96, P < 0.05) (Fig. 5b and Table 4). Univariate Cox regression analysis also indicated that stage II, stage III and squamous histologic type were poor prognostic factors for prediction of DFS, and stage II, stage III and stage IV were poor prognostic factors for prediction of OS (Table 4). However, subsequent multivariate regression analysis indicated that only stage II, stage III and risk score were independent prognostic factors for prediction of both DFS and OS (P < 0.05). These results suggest that the six-gene signature is valid and reliable across datasets and platforms.

**Table 4:** Univariate and multivariate Cox regression analysis of the gene signature and survival of NSCLC patients in TCGA cohort

Variables	Group	Patients (N)	Univariate analysis		Multivariate analysis	
			HR (95% CI)	*P*	HR (95% CI)	*P*
DFS						
Age	≤ 67/> 67	383/378	0.97 (0.75–1.26)	8.30E−01	1.11 (0.85–1.45)	4.30E−01
Gender	Female/male	326/435	0.96 (0.74–1.25)	7.60E−01	1.08 (0.81–1.43)	6.00E−01
Stage	I/II	410/220	1.64 (1.21–2.22)	1.30E−03	1.74 (1.28–2.37)	3.60E−04
Stage	I/III	410/109	2.16 (1.51–3.08)	2.20E−05	2.25 (1.57–3.22)	9.30E−06
Stage	I/IV	410/22	1.76 (0.82–3.80)	1.50E−01	1.56 (0.71–3.40)	2.70E−01
Histological type	Adeno/squamous	395/366	0.65 (0.50–0.85)	1.60E−03	0.58 (0.43–0.77)	1.60E−04
Risk score	Low/high	380/381	1.33 (1.02–1.73)	3.70E−02	1.40 (1.08–1.83)	1.30E−02
OS						
Age	≤ 67/> 67	383/378	1.25 (0.98–1.58)	7.10E−02	1.34 (1.05–1.70)	1.90E−02
Gender	Female/male	326/435	1.22 (0.96–1.57)	1.10E−01	1.19 (0.92–1.53)	2.00E−01
Stage	I/II	410/220	1.51 (1.14–2.00)	4.30E−03	1.45 (1.09–1.93)	1.10E−02
Stage	I/III	410/109	2.07 (1.50–2.85)	9.60E−06	2.04 (1.47–2.83)	1.80E−05
Stage	I/IV	410/22	2.77 (1.59–4.83)	3.20E−04	2.83 (1.60–5.00)	3.40E−04
Histological type	Adeno/squamous	395/366	1.13 (0.89–1.44)	3.20E−01	0.93 (0.71–1.22)	5.90E−01
Risk score	Low/high	380/381	1.54 (1.20–1.96)	5.50E−04	1.48 (1.14–1.91)	3.30E−03

HR: hazard ratio; CI: confidence interval; Adeno: adenocarcinoma; Squamous: squamous cell carcinoma

### The six-gene signature is association with several hallmarks

**Fig. 6:**
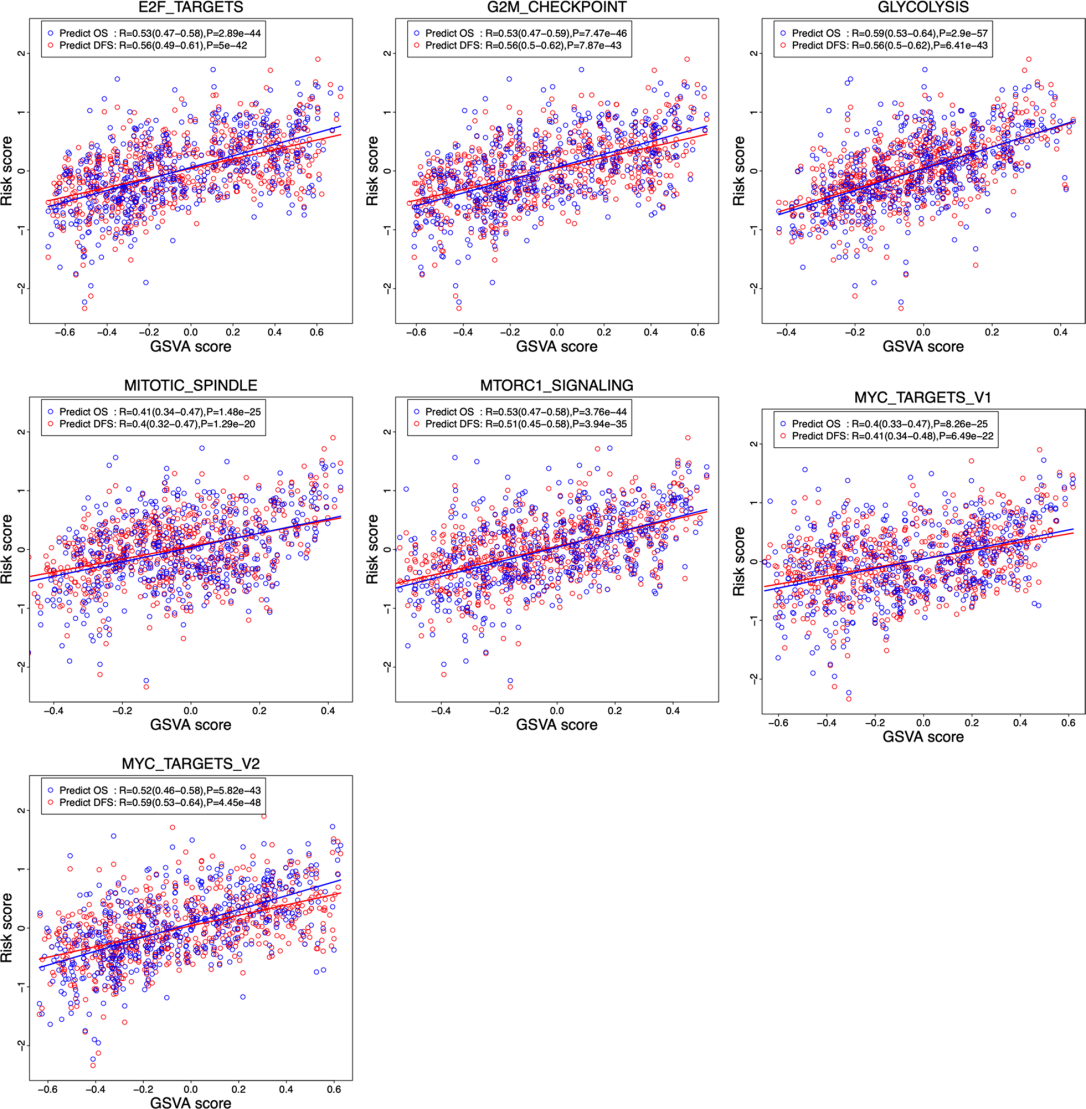
Association between the risk score of the six-gene prognosis signature and the GSVA score of 7 cancer hallmark gene sets

To identify the six-gene signature associated biological processes, the correlation of the risk score of the gene signature for predicting DFS/OS and the GSVA score of cancer hallmark gene sets was investigated. As shown in Fig. 6, a total of 7 hallmark gene sets (E2F_TARGETS, G2M_CHECKPOINT, GLYCOLYSIS, MITOTIC_SPINDLE, MTORC1-SIGNALING, MYC-TARGETS-V1, MYC-TARGETS-V2) were identified to be correlated with risk score [correlation coefficients (R) is higher than or equal to 0.4; P < 0.0001]. Interestingly, these biological processes and the risk score displayed the same trend, suggesting that activation of theses hallmarks might participate in the process of tumor progression and affect the survival of the patients with NSCLC.

## Discussion

Numerous reports have indicated that disturbed gene expression may be implicated in various aspects of tumor, including tumorigenesis, progression and prognosis [19–21]. Some genes have been considered as prospective biomarkers to predict prognosis in NSCLC patients [14, 22, 23]. However, several concerns limit their prognostic and predicative power, such as inadequate samples, lack of DFS prediction, and lack of effective validation. In this study, we developed and validated a novel prognostic six-gene signature that was found to be significantly associated with both the DFS and OS of NSCLC patients. Our results revealed that this classifier could successfully identify high-risk and low-risk NSCLC patients with significant differences in both DFS and OS. In addition, the prognostic value of the six-gene signature was verified in three GEO datasets and an independent TCGA dataset, suggesting the reproducibility and reliability of the six-gene signature for both DFS and OS prediction in NSCLC.

The clinical prognostic factors in NSCLC include stage, age, gender and performance status [6, 24]. Our study showed that stage and age were significantly correlated with both the DFS and OS of patients in GSE31210; performance status was significantly associated with DFS, and stage and performance status were related to OS of patients in GSE37745; stage and gender were significantly involved in OS of patients in GSE50081. In the entire patient cohort, stage was identified as an independent prognostic factor for DFS, and age and stage were associated with OS of NSCLC patients. Furthermore, we performed a stratification analysis on the entire cohort, and found that the prognostic power of the six-gene signature was independent of age, gender and stage. Interestingly, Birim et al. [24] indicated that non-squamous cell histology was a risk factor for postoperative outcome in NSCLC. Our study showed that histological type had no significant association with either DFS or OS in NSCLC. While stratification analysis indicated that high-risk group had significantly shorter DFS and OS than low-risk group for patient with adenocarcinoma but not squamous carcinoma.

Currently, tumor stage has been broadly utilized as a strong indicator of survival in NSCLC [25]. However, the current staging system is far from accurate in the aspect of survival prediction at the individual level [26]. As expected in our study, univariate and multivariate analysis showed that stage II, stage III, and stage IV were significantly associated with OS and DFS in the entire GEO cohort. However, stage II, and stage III were found to be the independent prognostic factors for prediction of both DFS and OS in the TCGA database. As documented, age is a main indicator of patient survival, and younger patients are tended to survive longer than the older ones [27–29]. Nevertheless, age alone is not a survival indicator for cancer patients because older patients are less likely to receive adjuvant therapy [30]. Multivariate analysis showed that age was significantly associated with OS in the entire GEO cohort and the TCGA cohort, but there was no correlation between DFS and age in these two cohorts. Thus, compared to age and stage, the risk score was a more reliable prognostic factor for NSCLC patients.

Several gene mutations have been revealed to be associated with the pathogenesis of NSCLC, such as *EGFR* and *KRAS* mutations [31]. Notably, *EGFR* or *KRAS* mutated lung cancer accounts for a significant subgroup of NSCLC, especially in adenocarcinoma [32, 33]. Markedly, targeting *EGFR* mutations has changed the therapeutic paradigm in NSCLC patients harboring *EGFR* mutations [34]. Over the last decade, multiple EGFR tyrosine kinase inhibitors (TKIs) have been developed to target mutated *EGFR*, and have achieved a better survival in patients with *EGFR* mutations than in those with the wild type [35]. Whereas, *KRAS* mutations predict a worse prognosis among NSCLC patients treated by chemotherapy and EGFR-TKIs [36, 37]. In the present study, univariate analysis indicated that *EGFR* mutations, but not *KRAS* mutations, were correlated with both DFS and OS, whereas multivariate analysis indicated that *EGFR* mutation status did not act as an independent prognostic factor.

In this study, we developed a prognostic six-gene signature for both DFS and OS prediction in NSCLC. Most of these genes have not been well characterized in tumor biology, except for *CDCP1*. CDCP1, also known as CUB domain-containing protein 1, is a transmembrane glycoprotein, whose phosphorylation is linked with the progression and metastasis of several cancers [38, 39]. In addition, blocking of CDCP1 has been shown to be a potential mode for therapeutic intervention against metastatic disease [38, 40]. Chiu et al. [41] showed that the ADAM9 metalloprotease enhanced CDCP1 expression via activating EGFR signaling pathways in advanced lung cancer disease. Ikeda et al. [42] revealed that CDCP1 expression was an independent prognostic factor for both OS and DFS, and could be used as an useful marker for survival prediction of patients with lung adenocarcinoma. Our study combined these 6 genes into a single panel, and established its prognostic value in both DFS and OS in NSCLC.

As reported, the complexity of cancer can be decreased and presented by a few cancer hallmarks that enable cancer cell proliferation and metastasis. These hallmarks can offer a framework to understand the cancer diversity. Hence, we focused on detecting the association of prognostic gene signature and cancer hallmarks [43]. Studies have revealed that DNA replication, cell cycle, DNA damage repair, apoptosis, chromosome and gene instability, energy supply play important roles in cancer progression [44–47]. Coincidentally, the GSVA results demonstrated that the six-gene signature was remarkably connected with these biological processes. Specifically, E2F targets have been demonstrated to participate in DNA replication, cell cycle, DNA damage repair, apoptosis [48]. G2M checkpoint, mitotic spindle, and MYC targets have been reported to contribute to the instability of chromosome and gene [49–51]. Utilization of glycolysis-related metabolic pathway has been implicated to provide ATP as a main source of energy supply for cancers [52]. Moreover, mTORC1 signaling has been suggested to be activated in human fibrolamellar liver carcinoma [53]. Demonstrated here, the results of present study demonstrated that the six-gene signature correlated with several cancer-progression associated biological processes which supported the DFS/OS predictive ability of the signature. Significantly, the correlation analysis in our study showed that patients having these activated biological processes tended to have adverse outcomes. Thus, this further confirmed that our six-gene signature used for predicting prognosis was reasonable and reliable.

In this work, some limitations need to be acknowledged. First, a few clinical characters presented an unbalanced distribution, such as an overwhelming majority of patients in stage I/II and presenting a histological adenocarcinoma type. Thus, the robustness of the six-gene signature requires further validation in large-scale prospective investments. Second, most of the genes identified here are rarely reported in the academic literature, and there are no experimental data regarding the identified signature, thus more evidence is needed to elucidate the inherent correlation between the six-gene signature and the prognosis of NSCLC patients. Despite these drawbacks, our results demonstrate valuable information on the importance and significance of the six-gene signature in both DFS and OS prediction in NSCLC.

## Conclusions

In this study, we developed an innovative six-gene prognostic signature for both DFS and OS prediction in NSCLC patients. The six-gene signature was an independent prognostic factor, and might complement clinicopathological factors and facilitate the personalized treatment of NSCLC patients. Large-scale prospective investments should be applied for further assessment of the robustness of this signature in future studies.

## Additional files

**Additional file 1: Table S1.** Validating the prediction power of the gene signature for OS in the combined GEO dataset by re-sampling analysis.

**Additional file 2: Table S2.** Validating the prediction power of the gene signature for DSF in the combined GEO dataset by re-sampling analysis.

## Data Availability

The datasets used and/or analyzed during the current study are available from the corresponding author on reasonable request.
