# Supplementary material for: A robust six-gene prognostic signature for prediction of both disease-free and overall survival in non-small cell lung cancer
Source: J Transl Med. 2019 May 14;17:152. doi: 10.1186/s12967-019-1899-y (PMC6515678; doi:10.1186/s12967-019-1899-y)
Supplement: Supplementary file 1 — Additional file 1: Table S1. Validating the prediction power of the gene signature for OS in the combined GEO dataset by re-sampling analysis. [file 12967_2019_1899_MOESM1_ESM.docx]

Table S1. Validating the prediction power of the gene signature for OS in the combined GEO dataset by re-sampling analysis

| **Test** | **Univariate Cox analysis** | | **Kaplan-Meier analysis** | **AUC of ROC curve** | | | | |
| --- | --- | --- | --- | --- | --- | --- | --- | --- |
|  | **HR** | **Cox P** | **Log Rank P** | **1-Years** | **2-Years** | **3-Years** | **4-Years** | **5-Years** |
| 1 | 2.95(2.12-4.11) | 1.42E-10 | 1.75E-11 | 0.669 | 0.687 | 0.711 | 0.698 | 0.715 |
| 2 | 2.37(1.74-3.24) | 5.88E-08 | 2.28E-08 | 0.688 | 0.702 | 0.685 | 0.662 | 0.688 |
| 3 | 2.77(2.01-3.80) | 3.38E-10 | 5.71E-11 | 0.697 | 0.715 | 0.718 | 0.708 | 0.725 |
| 4 | 2.90(2.08-4.05) | 3.74E-10 | 5.33E-11 | 0.688 | 0.701 | 0.699 | 0.694 | 0.703 |
| 5 | 2.78(2.01-3.84) | 5.37E-10 | 9.28E-11 | 0.655 | 0.705 | 0.701 | 0.693 | 0.701 |
| 6 | 3.04(2.17-4.28) | 1.48E-10 | 1.62E-11 | 0.719 | 0.716 | 0.716 | 0.713 | 0.716 |
| 7 | 3.79(2.70-5.34) | 1.91E-14 | 2.22E-16 | 0.727 | 0.722 | 0.720 | 0.709 | 0.725 |
| 8 | 3.00(2.17-4.13) | 2.09E-11 | 1.97E-12 | 0.738 | 0.721 | 0.708 | 0.694 | 0.708 |
| 9 | 2.55(1.88-3.48) | 2.27E-09 | 5.80E-10 | 0.687 | 0.701 | 0.691 | 0.682 | 0.698 |
| 10 | 2.80(2.01-3.90) | 9.93E-10 | 1.83E-10 | 0.684 | 0.718 | 0.721 | 0.707 | 0.715 |
| 11 | 3.19(2.25-4.52) | 8.17E-11 | 7.01E-12 | 0.719 | 0.719 | 0.729 | 0.703 | 0.718 |
| 12 | 3.07(2.21-4.28) | 2.57E-11 | 2.25E-12 | 0.697 | 0.713 | 0.709 | 0.697 | 0.708 |
| 13 | 2.53(1.85-3.46) | 6.14E-09 | 1.75E-09 | 0.692 | 0.703 | 0.702 | 0.684 | 0.697 |
| 14 | 2.57(1.87-3.55) | 8.35E-09 | 2.37E-09 | 0.745 | 0.735 | 0.729 | 0.698 | 0.706 |
| 15 | 2.59(1.87-3.59) | 9.43E-09 | 2.58E-09 | 0.677 | 0.688 | 0.695 | 0.671 | 0.693 |
| 16 | 2.68(1.93-3.72) | 4.26E-09 | 1.00E-09 | 0.703 | 0.713 | 0.722 | 0.692 | 0.702 |
| 17 | 3.25(2.32-4.56) | 8.64E-12 | 5.07E-13 | 0.701 | 0.711 | 0.723 | 0.717 | 0.731 |
| 18 | 3.16(2.27-4.40) | 8.44E-12 | 5.76E-13 | 0.732 | 0.734 | 0.738 | 0.728 | 0.736 |
| 19 | 2.84(2.05-3.93) | 2.80E-10 | 4.30E-11 | 0.698 | 0.724 | 0.700 | 0.693 | 0.712 |
| 20 | 3.25(2.32-4.54) | 5.38E-12 | 2.99E-13 | 0.677 | 0.719 | 0.710 | 0.703 | 0.726 |
| 21 | 2.60(1.90-3.56) | 2.15E-09 | 5.16E-10 | 0.691 | 0.698 | 0.689 | 0.675 | 0.696 |
| 22 | 2.91(2.10-4.04) | 1.83E-10 | 2.44E-11 | 0.717 | 0.737 | 0.724 | 0.700 | 0.716 |
| 23 | 3.29(2.33-4.63) | 1.00E-11 | 5.86E-13 | 0.731 | 0.718 | 0.728 | 0.711 | 0.728 |
| 24 | 3.05(2.21-4.20) | 1.18E-11 | 9.82E-13 | 0.730 | 0.736 | 0.718 | 0.699 | 0.722 |
| 25 | 2.87(2.08-3.96) | 1.33E-10 | 1.81E-11 | 0.681 | 0.709 | 0.723 | 0.712 | 0.717 |
| 26 | 2.59(1.90-3.53) | 2.02E-09 | 4.97E-10 | 0.685 | 0.701 | 0.708 | 0.682 | 0.702 |
| 27 | 2.54(1.84-3.50) | 1.23E-08 | 3.63E-09 | 0.705 | 0.719 | 0.702 | 0.682 | 0.693 |
| 28 | 2.63(1.93-3.60) | 1.37E-09 | 3.12E-10 | 0.707 | 0.716 | 0.709 | 0.684 | 0.701 |
| 29 | 2.95(2.15-4.05) | 2.27E-11 | 2.30E-12 | 0.714 | 0.734 | 0.724 | 0.709 | 0.721 |
| 30 | 2.70(1.96-3.72) | 1.04E-09 | 2.11E-10 | 0.691 | 0.711 | 0.708 | 0.674 | 0.677 |
| 31 | 3.00(2.15-4.18) | 8.64E-11 | 9.38E-12 | 0.691 | 0.716 | 0.716 | 0.695 | 0.713 |
| 32 | 2.61(1.91-3.57) | 2.09E-09 | 5.01E-10 | 0.703 | 0.705 | 0.707 | 0.695 | 0.707 |
| 33 | 2.60(1.87-3.61) | 1.35E-08 | 3.77E-09 | 0.686 | 0.707 | 0.711 | 0.694 | 0.714 |
| 34 | 2.91(2.10-4.03) | 1.48E-10 | 1.92E-11 | 0.680 | 0.697 | 0.719 | 0.706 | 0.710 |
| 35 | 2.72(1.98-3.74) | 5.73E-10 | 1.06E-10 | 0.699 | 0.716 | 0.727 | 0.699 | 0.720 |
| 36 | 3.02(2.16-4.22) | 8.88E-11 | 9.63E-12 | 0.741 | 0.722 | 0.737 | 0.710 | 0.726 |
| 37 | 2.85(2.06-3.94) | 2.15E-10 | 3.10E-11 | 0.697 | 0.707 | 0.712 | 0.707 | 0.709 |
| 38 | 2.85(2.05-3.95) | 3.43E-10 | 5.20E-11 | 0.696 | 0.727 | 0.708 | 0.696 | 0.702 |
| 39 | 2.69(1.97-3.68) | 4.55E-10 | 8.74E-11 | 0.686 | 0.698 | 0.708 | 0.69 | 0.707 |
| 40 | 2.62(1.89-3.65) | 1.01E-08 | 2.70E-09 | 0.684 | 0.695 | 0.698 | 0.681 | 0.694 |
| 41 | 3.44(2.43-4.86) | 2.39E-12 | 8.76E-14 | 0.767 | 0.754 | 0.750 | 0.722 | 0.720 |
| 42 | 2.94(2.12-4.08) | 1.09E-10 | 1.31E-11 | 0.710 | 0.716 | 0.724 | 0.707 | 0.729 |
| 43 | 2.65(1.91-3.66) | 4.68E-09 | 1.14E-09 | 0.719 | 0.711 | 0.695 | 0.681 | 0.696 |
| 44 | 2.56(1.86-3.51) | 6.13E-09 | 1.69E-09 | 0.692 | 0.705 | 0.716 | 0.696 | 0.703 |
| 45 | 2.84(2.06-3.90) | 1.31E-10 | 1.87E-11 | 0.709 | 0.699 | 0.710 | 0.707 | 0.727 |
| 46 | 2.84(2.03-3.96) | 8.73E-10 | 1.46E-10 | 0.715 | 0.718 | 0.711 | 0.694 | 0.717 |
| 47 | 2.79(2.04-3.81) | 1.45E-10 | 2.26E-11 | 0.698 | 0.700 | 0.704 | 0.696 | 0.709 |
| 48 | 2.61(1.88-3.63) | 1.06E-08 | 2.85E-09 | 0.691 | 0.716 | 0.698 | 0.687 | 0.698 |
| 49 | 3.30(2.39-4.56) | 4.98E-13 | 1.93E-14 | 0.717 | 0.720 | 0.729 | 0.723 | 0.730 |
| 50 | 2.65(1.92-3.65) | 2.37E-09 | 5.51E-10 | 0.726 | 0.726 | 0.726 | 0.703 | 0.702 |
| 51 | 2.92(2.12-4.01) | 4.82E-11 | 5.64E-12 | 0.710 | 0.721 | 0.710 | 0.702 | 0.700 |
| 52 | 3.27(2.36-4.53) | 1.33E-12 | 6.10E-14 | 0.728 | 0.739 | 0.719 | 0.712 | 0.734 |
| 53 | 2.82(2.03-3.92) | 7.09E-10 | 1.21E-10 | 0.658 | 0.673 | 0.686 | 0.681 | 0.702 |
| 54 | 2.55(1.86-3.48) | 4.54E-09 | 1.23E-09 | 0.688 | 0.715 | 0.695 | 0.676 | 0.693 |
| 55 | 2.65(1.92-3.65) | 2.58E-09 | 5.99E-10 | 0.704 | 0.718 | 0.714 | 0.703 | 0.716 |
| 56 | 3.02(2.17-4.20) | 5.62E-11 | 5.83E-12 | 0.731 | 0.731 | 0.731 | 0.705 | 0.722 |
| 57 | 2.21(1.64-2.98) | 2.36E-07 | 1.15E-07 | 0.689 | 0.706 | 0.714 | 0.695 | 0.703 |
| 58 | 2.39(1.76-3.26) | 2.96E-08 | 1.07E-08 | 0.689 | 0.697 | 0.705 | 0.677 | 0.697 |
| 59 | 2.86(2.09-3.91) | 5.90E-11 | 7.54E-12 | 0.726 | 0.720 | 0.710 | 0.690 | 0.703 |
| 60 | 2.86(2.08-3.93) | 8.75E-11 | 1.16E-11 | 0.733 | 0.724 | 0.723 | 0.698 | 0.716 |
| 61 | 3.20(2.30-4.46) | 6.92E-12 | 4.26E-13 | 0.701 | 0.715 | 0.725 | 0.692 | 0.713 |
| 62 | 3.10(2.23-4.30) | 1.28E-11 | 1.01E-12 | 0.712 | 0.715 | 0.704 | 0.689 | 0.700 |
| 63 | 2.59(1.88-3.55) | 4.91E-09 | 1.28E-09 | 0.698 | 0.719 | 0.704 | 0.680 | 0.692 |
| 64 | 2.85(2.06-3.93) | 1.84E-10 | 2.66E-11 | 0.740 | 0.724 | 0.725 | 0.704 | 0.717 |
| 65 | 2.74(2.00-3.76) | 4.24E-10 | 7.54E-11 | 0.713 | 0.723 | 0.708 | 0.692 | 0.704 |
| 66 | 2.81(2.03-3.88) | 3.67E-10 | 5.96E-11 | 0.683 | 0.706 | 0.700 | 0.682 | 0.702 |
| 67 | 2.55(1.86-3.50) | 7.37E-09 | 2.08E-09 | 0.747 | 0.742 | 0.710 | 0.684 | 0.691 |
| 68 | 2.33(1.70-3.20) | 1.50E-07 | 6.47E-08 | 0.697 | 0.700 | 0.702 | 0.681 | 0.689 |
| 69 | 2.81(2.04-3.87) | 2.98E-10 | 4.71E-11 | 0.726 | 0.732 | 0.709 | 0.695 | 0.711 |
| 70 | 2.63(1.92-3.62) | 2.30E-09 | 5.41E-10 | 0.708 | 0.715 | 0.709 | 0.688 | 0.708 |
| 71 | 2.13(1.56-2.91) | 2.19E-06 | 1.26E-06 | 0.665 | 0.698 | 0.708 | 0.691 | 0.698 |
| 72 | 2.48(1.80-3.42) | 2.48E-08 | 8.13E-09 | 0.711 | 0.712 | 0.705 | 0.691 | 0.705 |
| 73 | 2.75(1.99-3.79) | 8.71E-10 | 1.62E-10 | 0.704 | 0.714 | 0.716 | 0.700 | 0.713 |
| 74 | 2.53(1.85-3.45) | 5.79E-09 | 1.63E-09 | 0.669 | 0.690 | 0.693 | 0.669 | 0.689 |
| 75 | 3.42(2.48-4.73) | 7.23E-14 | 1.78E-15 | 0.719 | 0.735 | 0.726 | 0.717 | 0.730 |
| 76 | 3.36(2.4-04.69) | 1.28E-12 | 5.07E-14 | 0.762 | 0.749 | 0.733 | 0.712 | 0.720 |
| 77 | 2.49(1.81-3.41) | 1.80E-08 | 5.79E-09 | 0.739 | 0.728 | 0.701 | 0.666 | 0.682 |
| 78 | 2.39(1.76-3.25) | 2.38E-08 | 8.49E-09 | 0.675 | 0.691 | 0.697 | 0.673 | 0.697 |
| 79 | 2.85(2.06-3.96) | 3.40E-10 | 5.17E-11 | 0.695 | 0.712 | 0.694 | 0.702 | 0.718 |
| 80 | 2.46(1.82-3.33) | 5.83E-09 | 1.77E-09 | 0.701 | 0.725 | 0.695 | 0.667 | 0.683 |
| 81 | 3.50(2.48-4.93) | 8.85E-13 | 2.58E-14 | 0.689 | 0.719 | 0.716 | 0.710 | 0.717 |
| 82 | 3.48(2.51-4.83) | 8.75E-14 | 2.00E-15 | 0.707 | 0.708 | 0.719 | 0.705 | 0.716 |
| 83 | 2.64(1.91-3.66) | 4.87E-09 | 1.21E-09 | 0.685 | 0.700 | 0.714 | 0.692 | 0.705 |
| 84 | 3.13(2.26-4.34) | 5.83E-12 | 3.90E-13 | 0.734 | 0.743 | 0.734 | 0.716 | 0.726 |
| 85 | 3.48(2.46-4.91) | 1.34E-12 | 4.24E-14 | 0.709 | 0.725 | 0.708 | 0.684 | 0.699 |
| 86 | 3.16(2.28-4.38) | 4.97E-12 | 3.25E-13 | 0.741 | 0.740 | 0.720 | 0.706 | 0.730 |
| 87 | 2.81(2.03-3.88) | 4.66E-10 | 7.62E-11 | 0.715 | 0.730 | 0.719 | 0.706 | 0.706 |
| 88 | 2.73(1.96-3.82) | 3.61E-09 | 7.87E-10 | 0.695 | 0.682 | 0.697 | 0.680 | 0.686 |
| 89 | 2.65(1.89-3.70) | 1.23E-08 | 3.23E-09 | 0.705 | 0.700 | 0.693 | 0.686 | 0.695 |
| 90 | 2.41(1.77-3.29) | 2.70E-08 | 9.62E-09 | 0.686 | 0.701 | 0.703 | 0.684 | 0.701 |
| 91 | 3.05(2.18-4.26) | 5.70E-11 | 5.63E-12 | 0.718 | 0.715 | 0.717 | 0.696 | 0.702 |
| 92 | 3.00(2.20-4.08) | 3.34E-12 | 2.62E-13 | 0.716 | 0.720 | 0.732 | 0.728 | 0.739 |
| 93 | 3.27(2.37-4.52) | 6.53E-13 | 2.79E-14 | 0.733 | 0.726 | 0.739 | 0.713 | 0.727 |
| 94 | 3.31(2.35-4.65) | 6.28E-12 | 3.21E-13 | 0.709 | 0.710 | 0.697 | 0.688 | 0.711 |
| 95 | 2.60(1.89-3.58) | 5.46E-09 | 1.42E-09 | 0.681 | 0.704 | 0.712 | 0.692 | 0.702 |
| 96 | 2.95(2.12-4.09) | 1.08E-10 | 1.31E-11 | 0.727 | 0.725 | 0.714 | 0.697 | 0.710 |
| 97 | 2.82(2.04-3.91) | 4.38E-10 | 7.18E-11 | 0.683 | 0.715 | 0.707 | 0.685 | 0.707 |
| 98 | 2.54(1.84-3.50) | 1.13E-08 | 3.32E-09 | 0.685 | 0.713 | 0.701 | 0.686 | 0.700 |
| 99 | 2.46(1.80-3.38) | 2.23E-08 | 7.39E-09 | 0.663 | 0.691 | 0.688 | 0.672 | 0.692 |
| 100 | 2.60(1.88-3.58) | 5.78E-09 | 1.51E-09 | 0.724 | 0.717 | 0.712 | 0.689 | 0.696 |
