# Supplementary material for: A robust six-gene prognostic signature for prediction of both disease-free and overall survival in non-small cell lung cancer
Source: J Transl Med. 2019 May 14;17:152. doi: 10.1186/s12967-019-1899-y (PMC6515678; doi:10.1186/s12967-019-1899-y)
Supplement: Supplementary file 2 — Additional file 2: Table S2. Validating the prediction power of the gene signature for DSF in the combined GEO dataset by re-sampling analysis. [file 12967_2019_1899_MOESM2_ESM.docx]

Table S2. Validating the prediction power of the gene signature for DSF in the combined GEO dataset by re-sampling analysis

| **Test** | **Univariate Cox analysis** | | **Kaplan-Meier analysis** | **AUC of ROC curve** | | | | |
| --- | --- | --- | --- | --- | --- | --- | --- | --- |
|  | **HR** | **Cox P** | **Log Rank P** | **1-Years** | **2-Years** | **3-Years** | **4-Years** | **5-Years** |
| 1 | 2.64(1.77-3.93) | 1.92E-06 | 7.61E-07 | 0.691 | 0.665 | 0.664 | 0.674 | 0.687 |
| 2 | 2.27(1.53-3.37) | 4.73E-05 | 2.88E-05 | 0.702 | 0.667 | 0.645 | 0.678 | 0.690 |
| 3 | 3.33(2.22-4.98) | 5.75E-09 | 6.65E-10 | 0.700 | 0.678 | 0.668 | 0.691 | 0.708 |
| 4 | 2.27(1.52-3.37) | 5.56E-05 | 3.46E-05 | 0.684 | 0.654 | 0.658 | 0.662 | 0.681 |
| 5 | 2.98(2.00-4.45) | 8.97E-08 | 2.05E-08 | 0.739 | 0.702 | 0.685 | 0.690 | 0.704 |
| 6 | 2.50(1.70-3.67) | 3.38E-06 | 1.55E-06 | 0.721 | 0.681 | 0.662 | 0.657 | 0.685 |
| 7 | 2.42(1.62-3.60) | 1.34E-05 | 7.05E-06 | 0.666 | 0.655 | 0.634 | 0.634 | 0.665 |
| 8 | 2.73(1.85-4.04) | 4.85E-07 | 1.57E-07 | 0.725 | 0.667 | 0.665 | 0.687 | 0.700 |
| 9 | 2.54(1.74-3.71) | 1.42E-06 | 5.85E-07 | 0.734 | 0.712 | 0.676 | 0.685 | 0.690 |
| 10 | 2.59(1.74-3.84) | 2.30E-06 | 9.53E-07 | 0.711 | 0.685 | 0.665 | 0.675 | 0.695 |
| 11 | 2.36(1.60-3.48) | 1.53E-05 | 8.36E-06 | 0.685 | 0.668 | 0.651 | 0.650 | 0.670 |
| 12 | 3.28(2.19-4.91) | 8.27E-09 | 1.06E-09 | 0.727 | 0.695 | 0.676 | 0.689 | 0.714 |
| 13 | 2.22(1.53-3.23) | 3.15E-05 | 1.95E-05 | 0.700 | 0.660 | 0.656 | 0.648 | 0.680 |
| 14 | 2.40(1.64-3.52) | 6.93E-06 | 3.54E-06 | 0.717 | 0.670 | 0.653 | 0.650 | 0.676 |
| 15 | 3.17(2.07-4.87) | 1.29E-07 | 2.50E-08 | 0.703 | 0.682 | 0.672 | 0.683 | 0.701 |
| 16 | 2.51(1.68-3.73) | 5.90E-06 | 2.75E-06 | 0.674 | 0.647 | 0.635 | 0.641 | 0.673 |
| 17 | 2.47(1.66-3.67) | 8.50E-06 | 4.17E-06 | 0.730 | 0.674 | 0.662 | 0.670 | 0.688 |
| 18 | 2.69(1.84-3.94) | 3.39E-07 | 1.12E-07 | 0.708 | 0.665 | 0.644 | 0.648 | 0.665 |
| 19 | 3.05(2.03-4.59) | 7.12E-08 | 1.46E-08 | 0.717 | 0.672 | 0.667 | 0.679 | 0.703 |
| 20 | 2.25(1.54-3.27) | 2.57E-05 | 1.56E-05 | 0.698 | 0.646 | 0.648 | 0.639 | 0.668 |
| 21 | 2.80(1.88-4.18) | 4.09E-07 | 1.24E-07 | 0.745 | 0.679 | 0.656 | 0.664 | 0.688 |
| 22 | 2.94(1.99-4.32) | 5.15E-08 | 1.17E-08 | 0.732 | 0.705 | 0.694 | 0.710 | 0.721 |
| 23 | 2.29(1.55-3.39) | 3.18E-05 | 1.88E-05 | 0.681 | 0.661 | 0.653 | 0.650 | 0.664 |
| 24 | 2.84(1.89-4.27) | 5.33E-07 | 1.60E-07 | 0.715 | 0.682 | 0.670 | 0.692 | 0.711 |
| 25 | 2.37(1.61-3.49) | 1.23E-05 | 6.62E-06 | 0.720 | 0.684 | 0.653 | 0.673 | 0.690 |
| 26 | 2.36(1.60-3.50) | 1.76E-05 | 9.75E-06 | 0.699 | 0.669 | 0.646 | 0.658 | 0.683 |
| 27 | 2.82(1.90-4.21) | 3.41E-07 | 9.97E-08 | 0.713 | 0.675 | 0.651 | 0.672 | 0.693 |
| 28 | 2.40(1.64-3.51) | 7.08E-06 | 3.61E-06 | 0.683 | 0.665 | 0.661 | 0.672 | 0.680 |
| 29 | 2.91(1.96-4.32) | 1.30E-07 | 3.26E-08 | 0.717 | 0.673 | 0.659 | 0.669 | 0.700 |
| 30 | 2.81(1.90-4.16) | 2.68E-07 | 7.76E-08 | 0.714 | 0.691 | 0.680 | 0.679 | 0.695 |
| 31 | 2.48(1.70-3.63) | 2.60E-06 | 1.16E-06 | 0.691 | 0.647 | 0.631 | 0.637 | 0.671 |
| 32 | 2.19(1.49-3.21) | 6.97E-05 | 4.54E-05 | 0.699 | 0.650 | 0.619 | 0.627 | 0.643 |
| 33 | 2.49(1.68-3.69) | 5.72E-06 | 2.72E-06 | 0.659 | 0.655 | 0.638 | 0.660 | 0.685 |
| 34 | 2.38(1.61-3.52) | 1.32E-05 | 7.08E-06 | 0.698 | 0.692 | 0.672 | 0.671 | 0.681 |
| 35 | 3.11(2.07-4.66) | 4.07E-08 | 7.41E-09 | 0.739 | 0.679 | 0.673 | 0.688 | 0.711 |
| 36 | 2.41(1.63-3.58) | 1.16E-05 | 6.03E-06 | 0.691 | 0.666 | 0.662 | 0.673 | 0.685 |
| 37 | 2.41(1.62-3.58) | 1.27E-05 | 6.65E-06 | 0.662 | 0.662 | 0.643 | 0.648 | 0.671 |
| 38 | 2.61(1.77-3.85) | 1.36E-06 | 5.31E-07 | 0.737 | 0.674 | 0.662 | 0.690 | 0.695 |
| 39 | 3.27(2.20-4.88) | 5.26E-09 | 6.37E-10 | 0.725 | 0.686 | 0.674 | 0.678 | 0.704 |
| 40 | 2.63(1.77-3.89) | 1.45E-06 | 5.62E-07 | 0.693 | 0.668 | 0.656 | 0.670 | 0.700 |
| 41 | 2.83(1.91-4.21) | 2.68E-07 | 7.70E-08 | 0.695 | 0.666 | 0.662 | 0.672 | 0.688 |
| 42 | 2.73(1.83-4.08) | 8.72E-07 | 2.99E-07 | 0.720 | 0.649 | 0.648 | 0.678 | 0.715 |
| 43 | 3.08(2.02-4.69) | 1.57E-07 | 3.45E-08 | 0.733 | 0.715 | 0.704 | 0.722 | 0.731 |
| 44 | 2.38(1.59-3.58) | 2.86E-05 | 1.61E-05 | 0.706 | 0.645 | 0.637 | 0.643 | 0.667 |
| 45 | 2.08(1.40-3.08) | 2.59E-04 | 1.88E-04 | 0.696 | 0.675 | 0.631 | 0.639 | 0.644 |
| 46 | 3.05(2.02-4.62) | 1.36E-07 | 2.98E-08 | 0.778 | 0.716 | 0.700 | 0.704 | 0.722 |
| 47 | 2.55(1.71-3.80) | 3.86E-06 | 1.69E-06 | 0.661 | 0.653 | 0.625 | 0.648 | 0.663 |
| 48 | 2.93(1.95-4.40) | 2.42E-07 | 6.31E-08 | 0.713 | 0.687 | 0.671 | 0.676 | 0.701 |
| 49 | 2.23(1.51-3.31) | 6.31E-05 | 4.00E-05 | 0.709 | 0.669 | 0.633 | 0.637 | 0.663 |
| 50 | 2.38(1.61-3.51) | 1.42E-05 | 7.67E-06 | 0.682 | 0.655 | 0.630 | 0.663 | 0.684 |
| 51 | 2.91(1.93-4.38) | 3.00E-07 | 8.01E-08 | 0.719 | 0.683 | 0.693 | 0.693 | 0.710 |
| 52 | 2.52(1.73-3.68) | 1.55E-06 | 6.52E-07 | 0.683 | 0.656 | 0.657 | 0.674 | 0.699 |
| 53 | 2.92(1.94-4.38) | 2.54E-07 | 6.81E-08 | 0.739 | 0.707 | 0.707 | 0.720 | 0.728 |
| 54 | 3.08(2.01-4.71) | 2.36E-07 | 5.40E-08 | 0.702 | 0.689 | 0.672 | 0.701 | 0.732 |
| 55 | 2.98(2.01-4.42) | 5.27E-08 | 1.12E-08 | 0.689 | 0.681 | 0.682 | 0.690 | 0.709 |
| 56 | 2.74(1.84-4.07) | 6.17E-07 | 2.06E-07 | 0.747 | 0.698 | 0.676 | 0.691 | 0.707 |
| 57 | 2.60(1.72-3.93) | 6.48E-06 | 2.89E-06 | 0.714 | 0.698 | 0.669 | 0.670 | 0.681 |
| 58 | 2.79(1.87-4.15) | 4.28E-07 | 1.32E-07 | 0.725 | 0.665 | 0.648 | 0.673 | 0.691 |
| 59 | 2.75(1.82-4.16) | 1.46E-06 | 5.12E-07 | 0.765 | 0.687 | 0.689 | 0.693 | 0.715 |
| 60 | 2.99(1.99-4.51) | 1.45E-07 | 3.40E-08 | 0.723 | 0.677 | 0.668 | 0.690 | 0.703 |
| 61 | 2.82(1.91-4.19) | 2.32E-07 | 6.56E-08 | 0.721 | 0.677 | 0.656 | 0.663 | 0.691 |
| 62 | 2.18(1.50-3.16) | 4.33E-05 | 2.77E-05 | 0.692 | 0.641 | 0.628 | 0.633 | 0.673 |
| 63 | 2.61(1.79-3.80) | 5.34E-07 | 1.94E-07 | 0.692 | 0.654 | 0.649 | 0.672 | 0.701 |
| 64 | 2.91(1.97-4.32) | 9.54E-08 | 2.29E-08 | 0.676 | 0.681 | 0.648 | 0.679 | 0.694 |
| 65 | 2.77(1.88-4.09) | 3.06E-07 | 9.40E-08 | 0.703 | 0.683 | 0.661 | 0.673 | 0.704 |
| 66 | 2.85(1.93-4.23) | 1.75E-07 | 4.69E-08 | 0.707 | 0.668 | 0.671 | 0.674 | 0.702 |
| 67 | 3.11(2.09-4.64) | 2.43E-08 | 4.21E-09 | 0.732 | 0.689 | 0.665 | 0.685 | 0.712 |
| 68 | 2.42(1.64-3.57) | 7.62E-06 | 3.82E-06 | 0.686 | 0.672 | 0.659 | 0.682 | 0.700 |
| 69 | 2.36(1.61-3.46) | 1.17E-05 | 6.25E-06 | 0.722 | 0.680 | 0.661 | 0.665 | 0.694 |
| 70 | 1.88(1.30-2.74) | 9.03E-04 | 7.41E-04 | 0.674 | 0.644 | 0.636 | 0.621 | 0.637 |
| 71 | 2.90(1.92-4.39) | 4.44E-07 | 1.23E-07 | 0.724 | 0.671 | 0.663 | 0.682 | 0.692 |
| 72 | 2.90(1.92-4.37) | 3.80E-07 | 1.06E-07 | 0.711 | 0.694 | 0.679 | 0.719 | 0.732 |
| 73 | 2.98(1.98-4.48) | 1.78E-07 | 4.38E-08 | 0.724 | 0.706 | 0.684 | 0.691 | 0.720 |
| 74 | 2.83(1.92-4.18) | 1.45E-07 | 3.93E-08 | 0.716 | 0.683 | 0.691 | 0.706 | 0.718 |
| 75 | 2.49(1.68-3.68) | 4.67E-06 | 2.18E-06 | 0.723 | 0.682 | 0.666 | 0.654 | 0.672 |
| 76 | 2.14(1.45-3.16) | 1.26E-04 | 8.61E-05 | 0.662 | 0.651 | 0.650 | 0.655 | 0.679 |
| 77 | 3.14(2.10-4.69) | 2.29E-08 | 3.78E-09 | 0.755 | 0.694 | 0.695 | 0.716 | 0.718 |
| 78 | 3.46(2.28-5.26) | 5.78E-09 | 5.90E-10 | 0.703 | 0.688 | 0.679 | 0.685 | 0.709 |
| 79 | 2.87(1.91-4.31) | 3.68E-07 | 1.04E-07 | 0.735 | 0.705 | 0.694 | 0.704 | 0.706 |
| 80 | 2.06(1.42-2.98) | 1.27E-04 | 9.08E-05 | 0.681 | 0.655 | 0.636 | 0.649 | 0.678 |
| 81 | 3.12(2.07-4.71) | 5.70E-08 | 1.06E-08 | 0.750 | 0.710 | 0.687 | 0.690 | 0.716 |
| 82 | 2.59(1.76-3.81) | 1.45E-06 | 5.73E-07 | 0.678 | 0.662 | 0.649 | 0.664 | 0.678 |
| 83 | 2.77(1.86-4.11) | 4.98E-07 | 1.59E-07 | 0.729 | 0.691 | 0.676 | 0.682 | 0.712 |
| 84 | 2.56(1.76-3.73) | 9.18E-07 | 3.65E-07 | 0.690 | 0.663 | 0.645 | 0.644 | 0.652 |
| 85 | 3.45(2.27-5.24) | 6.78E-09 | 6.97E-10 | 0.729 | 0.686 | 0.698 | 0.721 | 0.734 |
| 86 | 3.11(2.05-4.72) | 9.24E-08 | 1.87E-08 | 0.740 | 0.707 | 0.686 | 0.695 | 0.716 |
| 87 | 3.03(2.04-4.50) | 3.98E-08 | 7.83E-09 | 0.707 | 0.677 | 0.666 | 0.699 | 0.717 |
| 88 | 2.65(1.77-3.95) | 1.98E-06 | 7.70E-07 | 0.723 | 0.655 | 0.653 | 0.677 | 0.703 |
| 89 | 2.15(1.46-3.18) | 1.07E-04 | 7.22E-05 | 0.711 | 0.655 | 0.635 | 0.627 | 0.649 |
| 90 | 2.67(1.82-3.93) | 6.18E-07 | 2.17E-07 | 0.689 | 0.684 | 0.689 | 0.707 | 0.708 |
| 91 | 3.36(2.20-5.12) | 1.75E-08 | 2.24E-09 | 0.693 | 0.667 | 0.662 | 0.696 | 0.722 |
| 92 | 2.73(1.83-4.08) | 9.61E-07 | 3.33E-07 | 0.715 | 0.676 | 0.671 | 0.670 | 0.690 |
| 93 | 3.71(2.41-5.71) | 2.42E-09 | 1.59E-10 | 0.740 | 0.700 | 0.689 | 0.723 | 0.734 |
| 94 | 2.45(1.66-3.62) | 6.55E-06 | 3.21E-06 | 0.722 | 0.694 | 0.674 | 0.679 | 0.698 |
| 95 | 2.86(1.92-4.24) | 1.92E-07 | 5.24E-08 | 0.680 | 0.682 | 0.670 | 0.690 | 0.719 |
| 96 | 2.33(1.60-3.39) | 9.63E-06 | 5.22E-06 | 0.689 | 0.671 | 0.655 | 0.670 | 0.690 |
| 97 | 2.73(1.82-4.09) | 1.20E-06 | 4.21E-07 | 0.709 | 0.668 | 0.661 | 0.679 | 0.705 |
| 98 | 2.63(1.76-3.93) | 2.56E-06 | 1.03E-06 | 0.693 | 0.668 | 0.656 | 0.668 | 0.695 |
| 99 | 2.72(1.81-4.09) | 1.42E-06 | 5.11E-07 | 0.688 | 0.661 | 0.637 | 0.645 | 0.669 |
| 100 | 2.25(1.54-3.30) | 3.00E-05 | 1.80E-05 | 0.653 | 0.616 | 0.618 | 0.636 | 0.660 |
